# Omission of Axillary Lymph Node Dissection in Patients with pT0-2 ER+/HER2− Breast Cancer with 3–5 Positive Lymph Nodes Undergoing Adjuvant Systemic Therapy and Radiation Does Not Impact Overall Survival: A National Cancer Database Analysis

**DOI:** 10.1245/s10434-025-18546-5

**Published:** 2025-10-24

**Authors:** Annie Tang, Peter S. Wu, Preeti Farmah, Katherine Schulz-Costello, Natalie Johnson, Veronica Jones, Jose Bazan, Jamie Rand

**Affiliations:** 1 Department of Surgery, City of Hope, Duarte, CA USA; 2 Department of Radiation Oncology, City of Hope, Duarte, CA USA

**Keywords:** SLNB, ALND, Omission, Axillary, Positive lymph nodes, Adjuvant

## Abstract

**Background:**

Recent trials established safety of axillary lymph node dissection (ALND) omission in patients with 1–2 positive lymph nodes (LN) on sentinel LN biopsy (SLNB). However, the benefit of ALND in patients with 3–5 positive LNs remains debated. We examined national trends of ALND versus SLNB in this subgroup and evaluated survival outcomes.

**Patients and Methods:**

Using the National Cancer Database, we identified patients with pT0–2 ER+/HER2− breast cancer with 3–5 positive LNs who underwent adjuvant chemotherapy, endocrine therapy, and radiation therapy from January 2012 to December 2020. We compared patients who had SLNB alone versus ALND ± SLNB

**Results:**

Among 13,270 patients, 1712 (12.9%) had SLNB and 11,558 (87.1%) had ALND. ALND rates decreased by 18.3% during the study period (93.4% to 75.1%). Compared with ALND, SLNB group had higher proportion of three positive LNs (63.1% versus 43.1%, *p* < 0.001), Charlson Comorbidity Index 0 (87.4% versus 84.4%, *p* = 0.001), pT1 tumor (42.8% versus 35.4%, *p* < 0.001), well-to-moderately differentiated tumor (72% versus 66.9%, *p* < 0.001), absence of lymphovascular invasion (42.7% versus 36.3%, *p* < 0.001), and lobular histology (16.5% versus 12.7%, *p* < 0.001). There was no difference in overall survival (OS) between SLNB and ALND in univariate or multivariable models (adjusted HR 1.0, *p* = 0.77).

**Conclusions:**

National rates for ALND decreased in patients with 3–5 positive LNs over the last decade. There was no difference in OS with omission of ALND in patients with ER+/HER2− breast cancer with 3–5 positive LNs, supporting further studies to evaluate deescalation of axillary surgery in this population.

Surgical treatment for breast cancer has evolved from radical mastectomies with complete axillary dissection toward deescalation of treatment. Axillary lymph node dissection (ALND) was routinely used to identify nodal disease and accomplish local control for several decades. Sentinel lymph node biopsy (SLNB) has since replaced ALND for axillary staging in patients with clinically node negative breast cancer.^1,2^ The trend toward SLNB has been fueled by the morbidity of ALND, most notably due to lymphedema risk. The cumulative incidence of lymphedema after ALND ranges from 3 to 36.7% with an average of 14.1%, and the addition of radiation to ALND can compound that risk toward the upper limits.^3-5^

In recent years, clinical practice guidelines have expanded to deescalate axillary surgery in patients with breast cancer with limited nodal involvement on the basis of studies showing similar outcomes with omission of ALND. The American College of Surgeons Oncology Group (ACOSOG) Z0011, Sentinel Node Biopsy Omission of Axillary Clearance After Macrometastases (SENOMAC), and International Breast Cancer Study Group (IBCSG) 23-01 randomized clinical trials established the safety of omission of ALND in patients with 1–2 positive lymph nodes on SLNB.^6-9^ These studies showed reduced morbidity with SLNB compared with ALND without compromising overall survival (OS). The After Mapping of the Axilla: Radiotherapy or Surgery (AMAROS) and Optimal Treatment of the Axilla – Surgery or Radiotherapy (OTOASOR) studies further established that regional nodal irradiation (RNI) in lieu of ALND after positive SLNB resulted in low axillary recurrence rates with no significant difference in locoregional control or survival.^10,11^ Current National Comprehensive Cancer Network (NCCN) guidelines state that no further axillary surgery is needed in patients who have cT1–T2, cN0 breast cancers with 1–2 positive sentinel lymph nodes who underwent upfront surgery with planned adjuvant radiation, whereas those with > 3 positive lymph nodes are recommended for completion ALND.^12^

In patients with more advanced nodal disease (> 3 positive lymph nodes) undergoing upfront surgery, the benefit of ALND remains unclear. There are limited data evaluating outcomes between SLNB and ALND in this patient population. The randomized clinical trials comparing SLNB and ALND in patients who underwent upfront surgery were limited mostly to 1–2 positive lymph nodes with a small percentage of > 3 positive lymph nodes.^10^ Retrospective data have been conflicting on whether there is an oncologic benefit of ALND in patients with > 3 positive lymph nodes.^13,14^ In addition, recent advancements in adjuvant systemic and radiation therapy may provide local and distant control while sparing the morbidity of ALND in select patient populations with locally advanced breast cancer.

Using the National Cancer Database (NCDB), we evaluated national trends in axillary surgery management in patients with pathologic T0–T2 estrogen receptor positive (ER+) human epidermal growth factor receptor 2 negative (HER2−) breast cancer with 3–5 positive lymph nodes who underwent surgery followed by adjuvant systemic and radiation treatments, and the impact of SLNB versus ALND on OS in this patient population.

## Patients and Methods

### Data Source

The NCDB is a hospital-based registry of cancer outcomes produced by the American Cancer Society and the American College of Surgeon’s Commission on Cancer (CoC). The deidentified database includes data collected from teaching hospitals, community cancer centers, and other cancer centers including Veterans’ Affairs (VA) hospitals located in 49 states and Puerto Rico. Only CoC approved hospitals are allowed to report data to the NCDB and an estimated 70% of all new diagnoses of cancer in the USA are captured by the database. Patients who have consultation and a treatment plan but do not undergo treatment are not included in the NCDB. This study was determined to be exempt from institutional board review on the basis of human subject study criteria at our corresponding institution.

### Study Population and Statistical Analysis

Women aged less than 75 years with ER+/HER− localized invasive breast cancer diagnosed from 2012 to 2020 with pathologic T0–T2 disease and 3–5 positive LN on surgical resection were queried from the NCDB. Exclusion criteria included: male gender; patients who received neoadjuvant chemotherapy; patients with > 1 primary breast cancer diagnosis; patients who did not receive adjuvant chemotherapy (CT), endocrine therapy (ET), and radiation (RT); and unknown type of LN surgery. To avoid survivorship bias, patients with fewer than 12 months of follow-up were excluded. The patients were divided into the following groups using the 2012 coding for LN surgery: (1) ALND (including ALND alone or SLNB + ALND) versus (2) SLNB alone.

Descriptive statistics (including frequency and percent) were calculated to characterize the study population. The patient variables collected included age, ethnicity, Charlson Comorbidity Index (CCI), facility type, insurance status, year of diagnosis, tumor grade, number of positive LN, number of nodes examined, lymphovascular invasion (LVI), surgical margins, progesterone receptor (PR) status, tumor histology, chemotherapy, endocrine therapy, and radiation treatment. The Cochran–Armitage test for trend was applied to assess whether the proportion of patients undergoing ALND versus SLNB changed significantly over time (by year).

A multivariable Cox regression was performed to determine the association of various demographic and pathologic factors, including type of axillary surgery, with OS. Demographic factors of interest were identified a priori: age (< 40, 40–49, 50–59, 60–69, 70–74 years), ethnoracial category (non-Hispanic white, non-Hispanic Black, non-Hispanic Asian/Pacific, Hispanic, other, unknown), performance status based on CCI (0, 1, 2+), insurance status (private, Medicare, Medicaid, other government, uninsured, unknown), pathologic grade (low, intermediate, high), PR status (positive, negative, unknown), pathologic T-stage (pT0/pTis/pT1mi, pT1a/b/c, pT2), LVI (absent, present, unknown), histology (ductal, lobular, mixed), and surgical margins (negative, positive, unknown). Patients with missing covariate variables and treatment data were excluded from the multivariable logistic regression model, while variables coded under the “unknown” category were included in the analysis. Variables with significant association with survival on univariate analysis were introduced in the multivariable model. Adjusted odds ratios (OR) with 95% confidence intervals (95% CI) are reported. All *p*-values are two-sided with statistical significance evaluated at the 0.05 alpha level.

Similarly, a multivariable logistic regression analysis was used to evaluate the association of demographic and prognostic factors with type of axillary surgery (ALND versus SLNB), coded as a binary variable. Variables with significant association with ALND on univariate analysis were introduced in the multivariable model. Adjusted OR with 95% CI are reported. All *p*-values are two-sided with statistical significance evaluated at the 0.05 alpha level. All analyses were performed using R version 44.11.2.

## Results

**Fig. 1:**
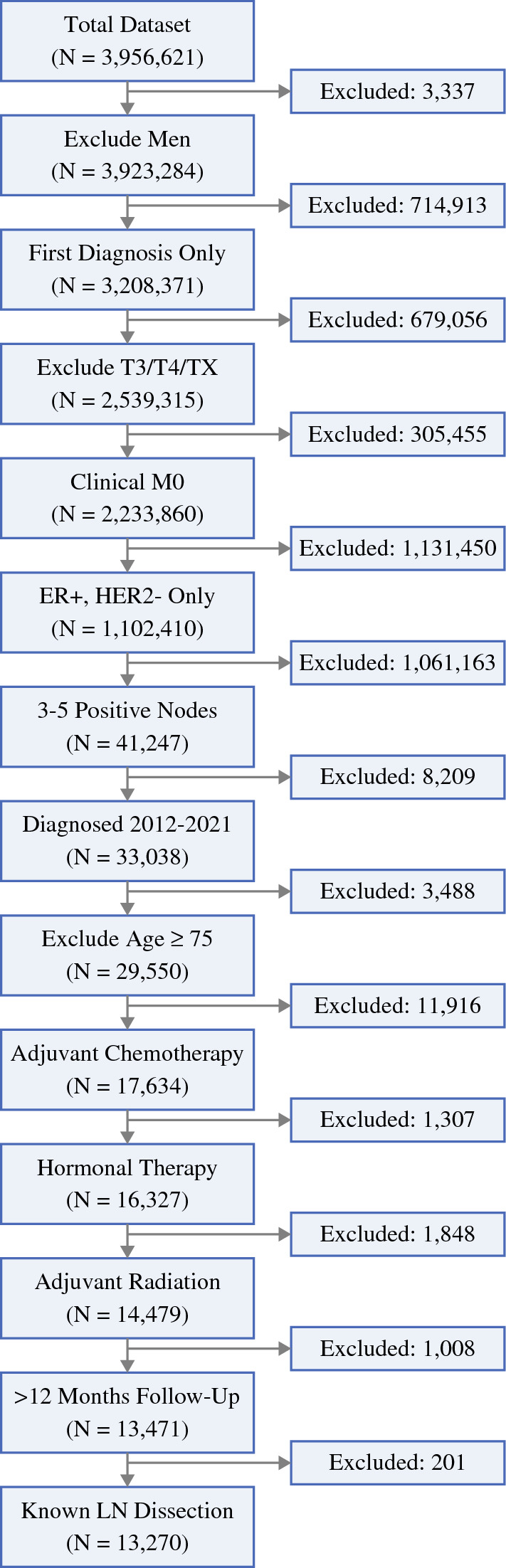
Consort flowchart showing patient selection from the NCDB for this study, patients were included if they had cT0–2 ER+ HER2− breast cancer diagnosed between 2012 and 2021 with 3–5 positive LN on surgical pathology and received adjuvant chemotherapy, endocrine therapy, and radiation therapy, and exclusion criteria included prior history of breast cancer, male gender, distant metastasis, age ≥ 75 years, unknown LN surgery, and follow up of ≤ 12 months; NCDB, National Cancer Database, ER, estrogen receptor; HER2, Herceptin epidermal growth factor receptor; LN, lymph node

The NCDB analysis identified 29,550 female patients under the age of 75 years who had pT0–2 ER+HER2− breast cancer with 3–5 positive lymph node on surgical pathology between 2012 and 2020. Of these patients, 14,479 (49.0%) completed adjuvant chemotherapy, endocrine therapy, and radiation therapy (Fig. 1). After excluding patients with less than 12 months of follow-up and patients with unknown LN surgery type, there were 13,720 patients included in the study. Of these, 1712 (12.9%) had SLNB and 11,558 (87.1%) had ALND (Fig. 1).

Most patients included in the study were non-Hispanic white (74.1%), > 50 years old (66.9%), CCI of 0 (84.8%), private insurance (65.6%), and were treated at a comprehensive community cancer program or academic program (37.1% and 28.1%, respectively). On pathology, most patients had three positive lymph nodes (45.6%), LVI present (50.6%), negative surgical margins (94%), pT2 disease (63%), moderately differentiated tumor grade (55.3%), PR-positive (66.5%), and ductal histology (77.4%). All patients in the study received adjuvant chemotherapy, endocrine therapy, and radiation.

**Fig. 2:**
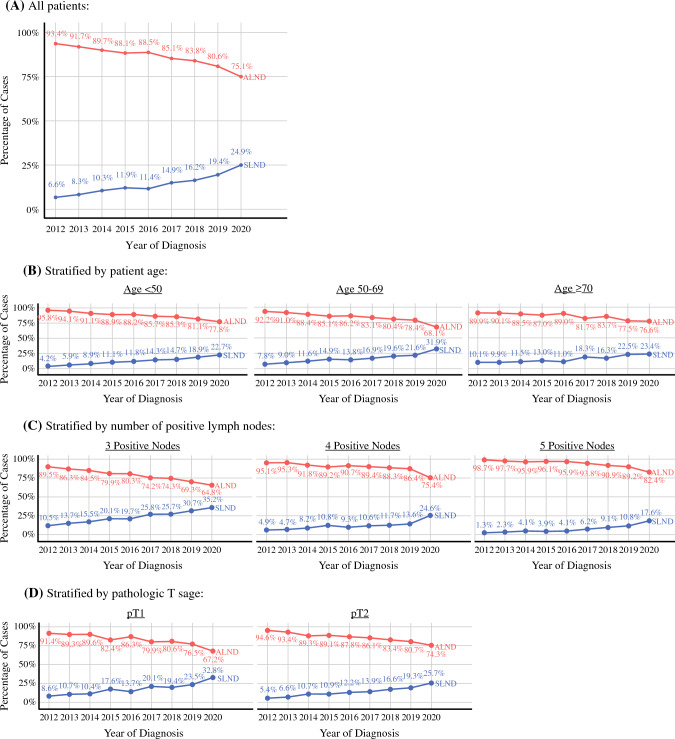
National trends in axillary surgery, including temporal trends in ALND and SLNB in patients with pT0–T2 ER+ HER2− breast cancer with 3–5 positive lymph nodes who underwent adjuvant systemic and radiation treatment; **A** there was a significant decrease in the proportion of patients who underwent ALND over time (Cochrane–Armitage *p* < 0.001); when stratified by age (**B**), number of positive lymph nodes (**C**), and pathologic T stage (**D**), there was a decrease in the percentage of patients who underwent ALND over time for all groups; ER, estrogen receptor; HER2, Herceptin epidermal growth factor receptor; ALND, axillary lymph node dissection; SLNB, sentinel lymph node biopsy

The proportion of patients who underwent ALND decreased by 18.3% during the study period, from 93.4% in 2012 to 75.1% in 2020 (Cochrane–Armitage *p* < 0.001) (Fig. 2A). When stratified by age (B), number of positive lymph nodes (C), and pathologic T stage (D), there was a decrease in the percentage of patients who underwent ALND over time for all groups (Fig. 2B–D). Compared with the ALND group, the SLNB group had a higher proportion of patients with three positive lymph nodes (63.1% versus 43.1%, *p* < 0.001), CCI score of 0 (87.4% versus 84.4%, *p* = 0.001), pT1 tumor (42.8% versus 35.4%, *p* < 0.001), well-to-moderately differentiated tumor (72% versus 66.9%, *p* < 0.001), absence of LVI (42.7% versus 36.3%, *p* < 0.001), negative surgical margins (94.3% versus 91.9%, *p* < 0.001), PR-positive (68.4% versus 54.1%, *p* < 0.001), and lobular histology (16.5% versus 12.7%, *p* < 0.001) (Table 1).

**Table 1:** Clinical characteristics of patients with ER+HER- breast cancer with 3–5 positive LNs who underwent adjuvant ET, CT, and RT; patients are stratified by type of axillary surgery: including all patients, ALND, or SLNB alone

Characteristics	Overall	ALND	SLNB	*p*-Value
	*N* = 13270	*N* = 11558	*N* = 1712	
**Age in** **years,** ***N*** **(%)**				**0.017**
< 40	1102 (8.3)	978 (8.5)	124 (7.2)	
40–49	3290 (24.8)	2909 (25.2)	381 (22.3)	
50–59	4221 (31.8)	3656 (31.6)	565 (33.0)	
60–69	3671 (27.7)	3161 (27.3)	510 (29.8)	
70–74	986 (7.4)	854 (7.4)	132 (7.7)	
**Positive** **regional** **nodes,** ***N*** **(%)**				**< 0.001**
3	6056 (45.6)	4976 (43.1)	1080 (63.1)	
4	4340 (32.7)	3912 (33.8)	428 (25.0)	
5	2874 (21.7)	2670 (23.1)	204 (11.9)	
**Ethnicity** **(%)**				0.124
White	9835 (74.1)	8542 (73.9)	1293 (75.5)	
Black	1527 (11.5)	1323 (11.4)	204 (11.9)	
Hispanic	982 (7.4)	866 (7.5)	116 (6.8)	
East Asian, South Asian, Pacific Islander	676 (5.1)	609 (5.3)	67 (3.9)	
Other	250 (1.9)	218 (1.9)	32 (1.9)	
**Charlson Comorbidity Index,** ***N *(%)**				**0.001**
0	11255 (84.8)	9758 (84.4)	1497 (87.4)	
1	1605 (12.1)	1422 (12.3)	183 (10.7)	
2+	410 (3.1)	378 (3.3)	32 (1.9)	
**Pathologic T stage,** ***N*** **(%)**				**< 0.001**
pT0 or pTis or pT1mi	79 (0.6)	70 (0.6)	9 (0.5)	
pT1a/b/c	4828 (36.4)	4095 (35.4)	733 (42.8)	
pT2	8363 (63.0)	7393 (64.0)	970 (56.7)	
**Facility type,** ***N* (%)**				0.213
Academic/research program	3734 (28.1)	3273 (28.3)	461 (26.9)	
Community cancer program	966 (7.3)	830 (7.2)	136 (7.9)	
Comprehensive community cancer program	4918 (37.1)	4263 (36.9)	655 (38.3)	
Integrated network cancer program	2550 (19.2)	2214 (19.2)	336 (19.6)	
Unknown	1102 (8.3)	978 (8.5)	124 (7.2)	
**Grade,** ***N*** **(%)**				**< 0.001**
Well differentiated	1631 (12.3)	1388 (12.0)	243 (14.2)	
Moderately differentiated	7338 (55.3)	6349 (54.9)	989 (57.8)	
Poorly differentiated	3817 (28.8)	3390 (29.3)	427 (24.9)	
Unknown	484 (3.6)	431 (3.7)	53 (3.1)	
**Lymphovascular invasion,** ***N*** **(%)**				**< 0.001**
Absent	4931 (37.2)	4200 (36.3)	731 (42.7)	
Present	6717 (50.6)	5942 (51.4)	775 (45.3)	
Unknown/indeterminate	1622 (12.2)	1416 (12.3)	206 (12.0)	
**Surgical margins,** ***N*** **(%)**				**< 0.001**
Negative	12473 (94.0)	10900 (94.3)	1573 (91.9)	
Positive macroscopic/microscopic	720 (5.4)	589 (5.1)	131 (7.7)	
Unknown	77 (0.7)	67 (0.6)	8 (0.5)	
**PR status,** ***N*** **(%)**				**< 0.001**
Positive	8831 (66.5)	7905 (68.4)	926 (54.1)	
Negative	915 (6.9)	826 (7.1)	89 (5.2)	
Unknown	3524 (26.6)	2827 (24.5)	697 (40.7)	
**Histology,** ***N*** **(%)**				**< 0.001**
Ductal	10271 (77.4)	8988 (77.8)	1283 (74.9)	
Lobular	1753 (13.2)	1470 (12.7)	283 (16.5)	
Mixed ductal and lobular	1246 (9.4)	1100 (9.5)	146 (8.5)	
**Year of diagnosis,** ***N*** **(%)**				**< 0.001**
2012	1807 (13.6)	1687 (14.6)	120 (7.0)	
2013	1731 (13.0)	1587 (13.7)	144 (8.4)	
2014	1595 (12.0)	1430 (12.4)	165 (9.6)	
2015	1541 (11.6)	1357 (11.7)	184 (10.7)	
2016	1603 (12.1)	1420 (12.3)	183 (10.7)	
2017	1471 (11.1)	1252 (10.8)	219 (12.8)	
2018	1287 (9.7)	1078 (9.3)	209 (12.2)	
2019	1240 (9.3)	1000 (8.7)	240 (14.0)	
2020	995 (7.5)	747 (6.5)	248 (14.5)	
**Insurance status,** ***N*** **(%)**				**0.015**
Private	8705 (65.6)	7557 (65.4)	1148 (67.1)	
Medicare	2723 (20.5)	2349 (20.3)	374 (21.8)	
Medicaid	1213 (9.1)	1093 (9.5)	120 (7.0)	
Uninsured	323 (2.4)	289 (2.5)	34 (2.0)	
Other government	170 (1.3)	151 (1.3)	19 (1.1)	
Unknown	136 (1.0)	119 (1.0)	17 (1.0)	
**Regional nodes examined > 10,** ***N*** **(%)**	9030 (68.0)	8719 (75.4)	311 (18.2)	**< 0.001**

ER, estrogen receptor; HER2, Herceptin epidermal growth factor receptor; LD, lymph node; ET, endocrine therapy; CT, chemotherapy; RT, radiation therapy; ALND, axillary lymph node dissection; SLNB, sentinel lymph node biopsy; PR, progesterone receptor

There was no significant difference in facility type between the ALND and SLNB groups. The SLNB group was more likely to have private insurance and less likely to have Medicaid (private insurance: 67.1% versus 65.4%, Medicaid: 7% versus 9.5%, *p* = 0.015).

**Fig. 3:**
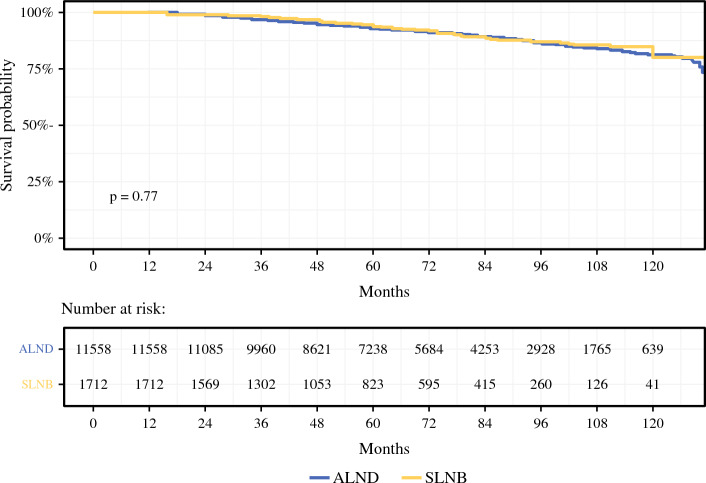
Kaplan–Meier survival curves comparing overall survival (OS) between patients who underwent ALND versus SLNB alone; there was no significant difference in OS between ALND and SLNB (*p* = 0.77); *ALND* axillary lymph node dissection, *SLNB* sentinel lymph node biopsy

**Fig. 4:**
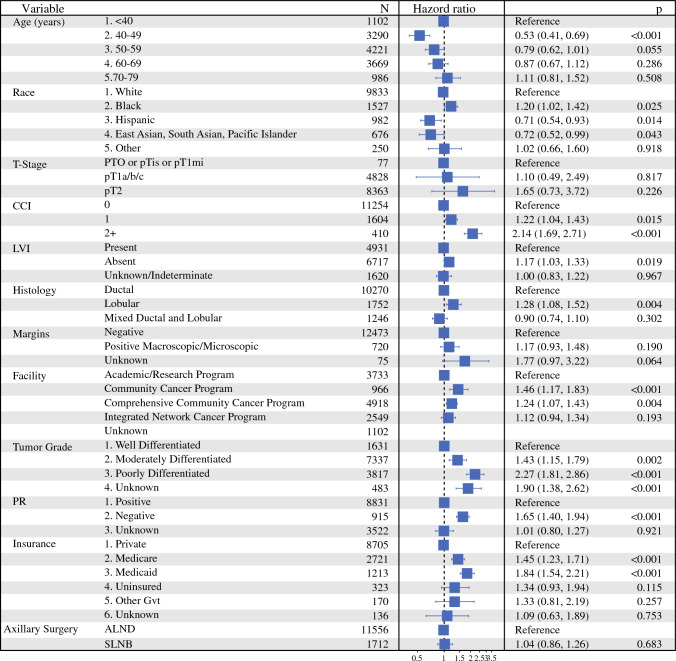
Multivariable Cox model of characteristics associated with OS; there was no significant difference in OS between SLNB and ALND, factors associated with improved OS included younger age (except < 40 years), Hispanic or Non-Hispanic Asian/Pacific, good performance status, private insurance, ductal histology, no LVI, PR-positive tumors, and well-differentiated disease; OS, overall survival; CCI, Charlson Comorbidity Index; LVI, lymphovascular invasion; PR, progesterone receptor; ALND, axillary lymph node dissection; SLNB, sentinel lymph node biopsy

The median follow-up was 73.5 months (95% CI 72.0–74.4). The 5-year OS was 93.9% (95% CI 93.4–94.3%) for the SLNB group and 94.4% (95% CI 93.1–95.8%) for the ALND group. There was no significant difference in OS between SLNB and ALND in patients with pT0–T2 ER+ HER2− breast cancer with 3–5 positive lymph nodes who underwent adjuvant chemotherapy, endocrine therapy, and radiation treatment on Kaplan–Meier analysis (Fig. 3). Similarly, there was no significant difference in OS between SLNB and ALND in univariate (HR 0.97, 95% CI 0.8–1.17, *p* = 0.768) or multivariable (HR 1.04, *p* = 0.7) Cox model analysis. Survival was better in patients with the following demographics: younger (except < 40 years), Hispanic or Non-Hispanic Asian/Pacific, good performance status, and private insurance. Pathologic features associated with improved survival on multivariable Cox model included: ductal histology, no LVI, PR-positive tumors, and well-differentiated disease (Fig. 4).

**Fig. 5:**
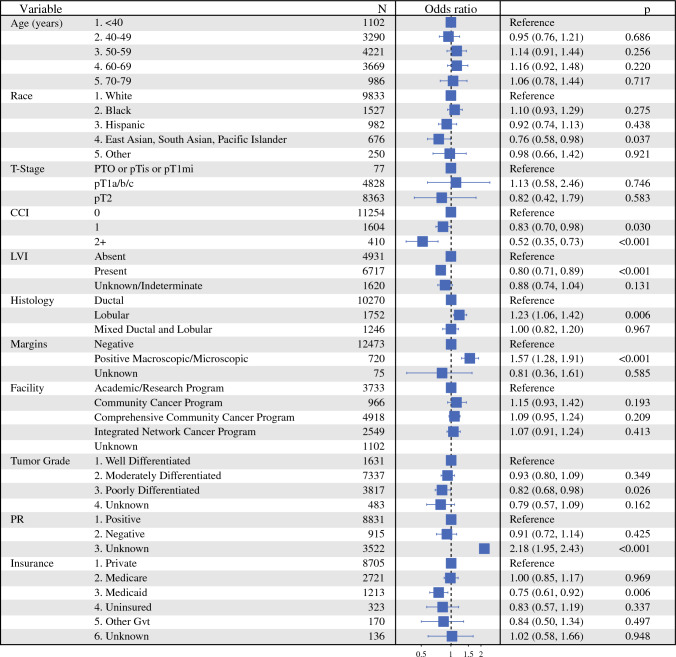
Multivariable logistic regression model of characteristics associated with SLNB when compared with ALND, factors associated with an increased likelihood of ALND included higher CCI, Medicaid insurance, LVI present, poorly differentiated tumor, ductal histology, and negative margins; OS, overall survival; CCI, Charlson Comorbidity Index; LVI, lymphovascular invasion; PR, progesterone receptor; ALND, axillary lymph node dissection; SLNB, sentinel lymph node biopsy

Finally, on multivariable logistic regression model of characteristics associated with type of axillary surgery, patients were more likely to undergo ALND if they had: higher CCI, Medicaid insurance, LVI present, poorly differentiated tumor, ductal histology, and negative margins (*p* < 0.001). Factors that were not significantly associated with type of axillary surgery included age, race, T-stage, and facility type (Fig. 5).

## Discussion

Despite the National Comprehensive Cancer Network (NCCN) guidelines recommending ALND in the setting of > 3 positive lymph nodes in patients with breast cancer undergoing upfront surgery, national rates for ALND have decreased in patients with pT0–T2 ER+ HER− breast cancer and 3–5 positive lymph nodes. Between 2012 and 2020, there was an 18.3% decrease in ALND, from 93.4% to 75.1%. NCDB analysis of this high-risk population that underwent adjuvant systemic therapy and radiation showed no significant difference in OS between SLNB and ALND.

The increase in omission of ALND observed in our review aligns with the overall trend of ALND omission in axillary management over the past 15 years. The ACOSOG Z0011 trial, which was published in 2010, showed no difference in survival with ALND omission for patients with cT1–2 N0 tumors with 1–2 positive LNs on SLNB who underwent breast conserving surgery followed by adjuvant whole breast radiation. Since the time of its publication, ALND in patients with 1–2 positive lymph nodes undergoing breast-conserving surgery decreased in Europe from 89% in 2010 to 46% in 2016, and in the USA from 42% in 2004 to 18% in 2016.^15,16^ Since that time, the criteria for safe omission of ALND has been expanded.

For example, in addition to those undergoing breast conservation, the AMAROS, OTOASOR, and SENOMAC trials established the safety of ALND omission in patients with limited LN involvement who were undergoing mastectomy and post-mastectomy radiation. Following these publications, ALND decreased from 47% in 2012 to 18% in 2020 in the USA in patients with limited LN involvement undergoing mastectomy, according to an NCDB analysis.^17^ When further expanding the criteria beyond Z0011, a review of NCDB data showed that ALND also decreased for patients with T3/T4 disease who otherwise met Z0011 criteria, from 54% in 2006 to 14% in 2016.^18^ Additionally, an NCDB analysis of patients with T1–3 breast cancer with > 3 positive lymph nodes who were treated between 2018 and 2020 similarly found that ALND rates decreased over the study period despite not fitting all the criteria for Z0011.^19^ ALND in this patient population decreased from 71% in 2018 to 59% in 2020.

Here, we sought to answer the question regarding trends of axillary surgery in patients with pT0–2 ER+/HER2− breast cancers who received adjuvant chemotherapy, endocrine therapy, and radiation, and whether the type of axillary surgery performed had an impact on OS in this patient population. Although the ALND rates in our study also significantly decreased throughout the study period, the ALND rates were higher than those reported in other studies. This is likely due to selection of a more specific patient population of pT0–T2 with 3–5 positive LN who underwent adjuvant chemotherapy, endocrine therapy, and radiation treatment. Additionally, the definition of ALND in NCDB studies has differed between different studies. We used the 2012 coding for LN surgery, as has been used in other studies looking at trends in axillary management.^17^ Limitations to NCDB analysis include reliance on accurate coding upon data input, which is subject to errors.

To better identify a subgroup of patients with locally advanced breast cancer who may benefit from deescalation of axillary surgery, our study included ER+ HER− patients who received recommended adjuvant treatments. During the study period between 2012 and 2020, both chemotherapy and endocrine therapy were standard-of-care systemic therapy in patients with 3–5 positive lymph nodes. Therefore, we included patients who underwent both systemic chemotherapy and endocrine therapy. In December 2021, the RxPONDER clinical trial established the role for the 21-Gene Assay to Inform Chemotherapy Benefit in Node-Positive Breast Cancer. The RxPonder trial showed that chemotherapy can be safely omitted in postmenopausal patients with 1–3 positive lymph nodes with a low genomic risk score who will receive adjuvant endocrine therapy; however, systemic chemotherapy is still recommended for patients with ≥ 4 positive lymph nodes.^20^ Optimal systemic therapy regimens will likely continue to evolve as novel therapeutics, such as cyclin dependent kinase (CDK) 4/6 inhibitors, are used more frequently in patients with high-risk ER+ HER2 negative cancers. As systemic therapies continue to improve, this may allow for further deescalation in the areas of surgery and/or radiation.

Regarding adjuvant radiation, we excluded patients who did not undergo adjuvant radiation for their locally advanced cancer. The NCCN guidelines recommend to “strongly consider” adjuvant radiation in patients with 1–3 positive lymph nodes, and that radiation is indicated in patients with ≥ 4 positive lymph nodes;^12^ therefore, adjuvant radiation is indicated in this population, regardless of whether patients underwent breast-conserving surgery or mastectomy. The AMAROS clinical trial showed that RNI in lieu of ALND after positive SLNB resulted in low axillary recurrence rates with no significant difference in locoregional control or survival.^10^ Therefore, we felt that adjuvant radiation was an important requirement when comparing outcomes and trends in patients with locally advanced cancers undergoing deescalation of axillary surgical management. Additional studies are warranted to evaluate whether RNI without further axillary dissection provides adequate locoregional control in patients with increased axillary tumor burden. Despite a lack of large, randomized control trials confirming oncologic safety in patients with 3–5 positive lymph nodes, ALND rates continue to decrease for patients with more advanced disease than those who meet the Z0011 criteria. Improvements in prognostic testing and treatment may contribute to this trend. Genomic testing has expanded and become more readily accessible to better guide benefit of adjuvant systemic treatment for patients with ER+ breast cancer.^20–22^ Thus, axillary staging may have less significance in deciding systemic treatment than genomic testing for many patients.^23^ In addition, there have been improvements of tailored systemic treatments with immunotherapy and precision oncology that have improved OS in select patients. With the introduction of CDK4/6 inhibitors for patients with early breast cancer, with criteria of either ≥ 4 positive lymph nodes or 1–3 positive lymph nodes with high-risk cancer features, a multidisciplinary approach is still needed for discussion of ALND omission.^24^ Lastly, radiation techniques have improved with potentially less morbidity than ALND. Wang et al. found that for patients with pT1–2 cancers with 1–2 positive lymph nodes who underwent mastectomy, ALND decreased from 47% to 18% while PMRT increased from 9.8% to 36.8%.^17^ The AMAROS study further established similar axillary control between ALND and regional nodal irradiation in patients with T1–2 breast cancers with positive sentinel lymph nodes and found less morbidity with axillary radiation. The study included 71 patients (5%) who had > 3 positive lymph nodes.^10^ A recent pooled meta-analysis of 145,548 patients showed no difference in OS, disease-free survival, locoregional recurrence, or axillary recurrence between SLNB alone, ALND, or axillary radiation groups, further supporting deescalation of axillary treatments.^25^

In our retrospective review of patients with pT0–T2 ER+ HER2− breast cancer with 3–5 positive lymph nodes who underwent adjuvant chemotherapy, endocrine therapy, and radiation treatment, we found no difference in OS between patients who underwent SLNB and ALND. In a Surveillance, Epidemiology, and End Results (SEER) database review from 2003 to 2008 of 9521 patients with T1–2 breast cancer with > 3 positive lymph nodes, Bonneau et al. similarly found no difference in OS and disease-specific survival between SLNB and ALND.^13^ In contrast, another review of the SEER database from 2000 to 2016 found that patients with T1–T2 breast cancers with 3–5 nodal metastases who underwent breast-conserving surgery had improved long-term survival with ALND compared with SLNB (HR 0.73).^14^ The contrasting data may be secondary to varying patient and clinical inclusion criteria of these studies. To better identify a subgroup of patients who may benefit from ALND omission, our study included ER+ HER− patients who received adjuvant chemotherapy, endocrine, and radiation treatment. This suggests that there may be a select patient population who may benefit from ALND and highlights the importance of adjuvant therapy in conjunction with local surgical control. In a subset analysis, we found that survival was better in patients who were younger (except < 40 years), Hispanic or non-Hispanic Asian/Pacific, had good performance status, had private insurance, ductal histology, no LVI, PR-positive tumors, and well-differentiated disease. Additional prospective studies are warranted to further delineate which patient population may benefit from ALND.

When comparing SLNB versus ALND in patients with ER+ HER2− breast cancer with 3–5 positive lymph nodes, we found that patients in the SLNB group were more likely to be > 50 years, have 3 positive lymph nodes, lower CCI, grade 1–2 disease, no LVI, and lobular histology. In a SEER database analysis of patients with 3–5 positive lymph nodes, the SLNB cohort similarly tended to be older and had a higher proportion of 3 positive LNs.^14^

We included patients with pT0, pTis, or pT1mic tumors, which together made up less than 1% of patients in this study (*n* = 79). This population includes patients with occult primary breast cancers (OPBC). OPBC is an important patient population in which surgical management is debated and the ability to deescalate surgery is being discussed. Of the 79 patients in our study, the majority underwent ALND (89%). Similar to the temporal trend seen in our study, a NCDB review by LaBella et al. studying patients with cT0N1–3 breast cancer found that there was a significant increase in percentage of patients with OPBC who underwent SLNB alone during the study period, from 4.7% in 2012 to 16.2% in 2021.^26^ Additional randomized controlled trials are needed to determine optimal axillary management in patients with OPBC.

Our study has several limitations. First, this is a retrospective review comparing two cohorts that were not matched. Additional randomized controlled trials are needed to better evaluate whether SLNB alone has similar oncologic outcomes compared with ALND. In addition, prospective studies may provide further evidence as to whether there are specific subpopulations who may derive benefit from ALND. Second, studies of ALND versus SLNB use different definitions of each surgery type. Given that NCDB allows for surgeons to self-designate lymph node sampling type, it is limited by subjectivity of the surgeon.^27^ Third, NCDB data lack disease recurrence information, thus differences in axillary recurrence and distant recurrence between the two groups could not be analyzed. We used OS as a method of analyzing outcomes in each group, but further studies are needed to evaluate effect of ALND omission in this population on axillary recurrence, distant recurrence, and disease-free survival. Fourth, the NCDB does not differentiate between micrometastasis and macrometastasis when assigning the number of positive nodes, as both are counted as positive. Therefore, it is possible that ALND was more likely to be omitted if patients had a combination of micrometastsis and macrometastasis, versus 3–5 macrometastasis. Further studies are needed to evaluate benefit of ALND on the basis of extent of axillary nodal involvement. Finally, national databases are limited to the data at hand and lack detailed information on specific treatment modalities, detailed tumor biology, and surgeon factors that may play into the decision of ALND versus SLNB.

In conclusion, we conducted a retrospective cohort study evaluating trends and survival outcomes between ALND and SLNB in patients with ER+ HER2− breast cancer with 3–5 positive lymph nodes who underwent adjuvant systemic and radiation treatment. Although there are limited data on ALND omission in this patient population, the national rate of ALND decreased by 18.3% between 2012 and 2020. In our review, there was no significant OS difference between those who underwent ALND and SLNB. This supports further studies to evaluate deescalation of axillary surgery in carefully selected patients who receive adjuvant radiation and systemic therapy. Further prospective and randomized controlled studies are warranted to assess the role of ALND in patients with more extensive axillary disease and to determine which patient populations may benefit from ALND when compared with SLNB.
